# Commentary: Phyllostomid bat microbiome composition is associated to host phylogeny and feeding strategies

**DOI:** 10.3389/fmicb.2018.02863

**Published:** 2018-11-22

**Authors:** Arinjay Banerjee, Edel Pérez-López, Karen Mossman

**Affiliations:** ^1^Department of Pathology and Molecular Medicine, Faculty of Health Sciences, Michael DeGroote Centre for Learning and Discovery, McMaster University, Hamilton, ON, Canada; ^2^Department of Biology, University of Saskatchewan, Saskatoon, SK, Canada

**Keywords:** bat, microbiome, sentinel, phytoplasma, plant pathogen

In their article, Carrillo-Araujo et al. show that Phyllostomidae (New World leaf-nosed bat family) gut microbiome composition is closely associated with host phylogeny. They provide evidence that feeding-strategy plays a role in the microbiome composition of these bats (Carrillo-Araujo et al., 2015). We were particularly intrigued by their detection of deoxyribonucleic acid (DNA) from *Firmicutes* in intestinal sections from these bats. The authors use a previously published approach of sequencing 16s rRNA for taxonomic assignment of the microbial community (Caporaso et al., 2012). 16s rRNA is a good predictor of taxonomic classification (Mizrahi-Man et al., 2013; Chaudhary et al., 2015), but it is not foolproof (reviewed here by Janda and Abbott, 2007). Data on the probability of correct taxonomic assignment will bolster the findings in this article and allow researchers to confidently design follow-up studies. Although not necessary for this study, testing a subset of the microbial population using an alternate sequencing-taxonomic grouping pipeline would further substantiate the taxonomic classification of microbial communities.

*Mollicutes* (Phylum *Firmicutes*) are a class of microorganisms that include phytoplasmas (‘*Candidatus* Phytoplasma’) that are being increasingly recognized for their role in plant diseases such as sapodilla little leaf, yellow leaf roll disease of peach, strawberry green petal and sugarcane white leaf syndrome. These diseases affect a diverse array of economically and ecologically important plant hosts around the world (Vesterinen et al., 2013; Pérez-López et al., 2016, 2017). *Mollicutes* are highly diverse and are made up of five orders, Acholeplasmatales, Anaeroplasmatales, Entoplasmatales, Haloplasmatales, and Mycoplasmatales. All members of these orders are obligate parasites. Genera ‘*Candidatus* Phytoplasma’ and *Spiroplasma*, from the Order Acholeplasmatales and Entoplasmatales, respectively, consist of plant pathogens. ‘*Candidatus* Phytoplasma’ and *Spiroplasma* represent 18% of the Class *Mollicutes* (Zhao et al., 2015).

‘*Candidatus* Phytoplasma’ consists of over 40 species that are known to be pathogenic in plants (Miyazaki et al., 2018). In the Genus *Spiroplasma*, at least two species have been identified as plant pathogens, *Spiroplasma citri*, the causative agent of citrus stubborn disease (Saglio et al., 1973) and *Spiroplasma kunkelii*, which is associated with corn stunt disease (Whitcomb et al., 1986).

In their article, the authors indicate that *Artibeus jamaicensis* have the highest relative abundance of *Mollicutes* in their intestinal contents (Carrillo-Araujo et al., 2015). In their article, supplementary Table S1 also indicates the presence of nucleic acids from *Firmicutes* in all bat species that were sampled. Looking at their data from a plant disease perspective, we wondered if frugivorous and nectivorous bats could play a role in the transmission of plant pathogens. Could this data set and the sampling methods established allow us to monitor bats as sentinels of plant pathogens? Here, we speculate upon the role of frugivorous and nectivorous bats as possible vectors of plant pathogens. We outline limitations of this study that do not allow us to fully establish the dynamics of plant pathogen-bat interactions. We also discuss future directions to firmly establish the role of bats as potential vectors of plant pathogens.

Recently, bats have been implicated as the reservoirs of several emerging viruses that cause serious disease in humans and agricultural animals (Calisher et al., 2006; Moratelli and Calisher, 2015; Zhou et al., 2018). These viruses fail to cause disease symptoms in experimentally or naturally infected bats (Munster et al., 2016; Hu et al., 2017; Schuh et al., 2017). We and others have since identified several adaptations in innate immune signaling molecules that might allow bats to control virus propagation more effectively than other mammals (Zhou et al., 2016; Banerjee et al., 2017; Xie et al., 2018). Bats are also recognized as potential reservoirs of pathogenic bacteria (Loftis et al., 2005; Becker et al., 2018). Plant pathogens do not generally infect mammalian hosts, but after careful analysis of the data in Carrillo-Araujo et al.'s article (Carrillo-Araujo et al., 2015), the role of bats as potential vectors/carriers of plant pathogens cannot be ruled out. We compared the extent of spread of phytoplasmas in the Americas (Figure 1A) and observed that it overlapped with the spread of frugivorous bats *A. jamaicensis* (Figure 1B) and *Carollia perspicillata* (Figure 1C) and nectivorous bats *Leptonycteris yerbabuenae* (Figure 1D) and *Glossophaga soricina* (Figure 1E) that were sampled by the authors. The authors mention that frugivorous and nectivorous bats diverged 20–18 million years ago (MYA). Evolutionary reconstructions show that the divergence of *Mollicutes* into two major branches occurred about 470 MYA, placing phytoplasmas and their closest ancestor, *Ancholeplasma* in the same branch (Maniloff, 2002). There is further evidence that phytoplasmas diverged from an *Acholeplasma*-like ancestor around 99 MYA (Zhao et al., 2015). Thus, phytoplasmas and frugivorous bats have co-existed for at least 18 million years. Could bats have acquired phytoplasmas as part of their microbiome after their divergence into frugivorous and nectivorous bats? Could bats have played a role in the spread of phytoplasmas? We do not know. Phytoplasmas have been detected in fruits in the United States and Canada (Bagadia et al., 2013; Rosete et al., 2015). There is a need to sample additional bat species to fully elucidate the overlap in the spread of phytoplasmas and frugivorous bats.

**Figure 1:**
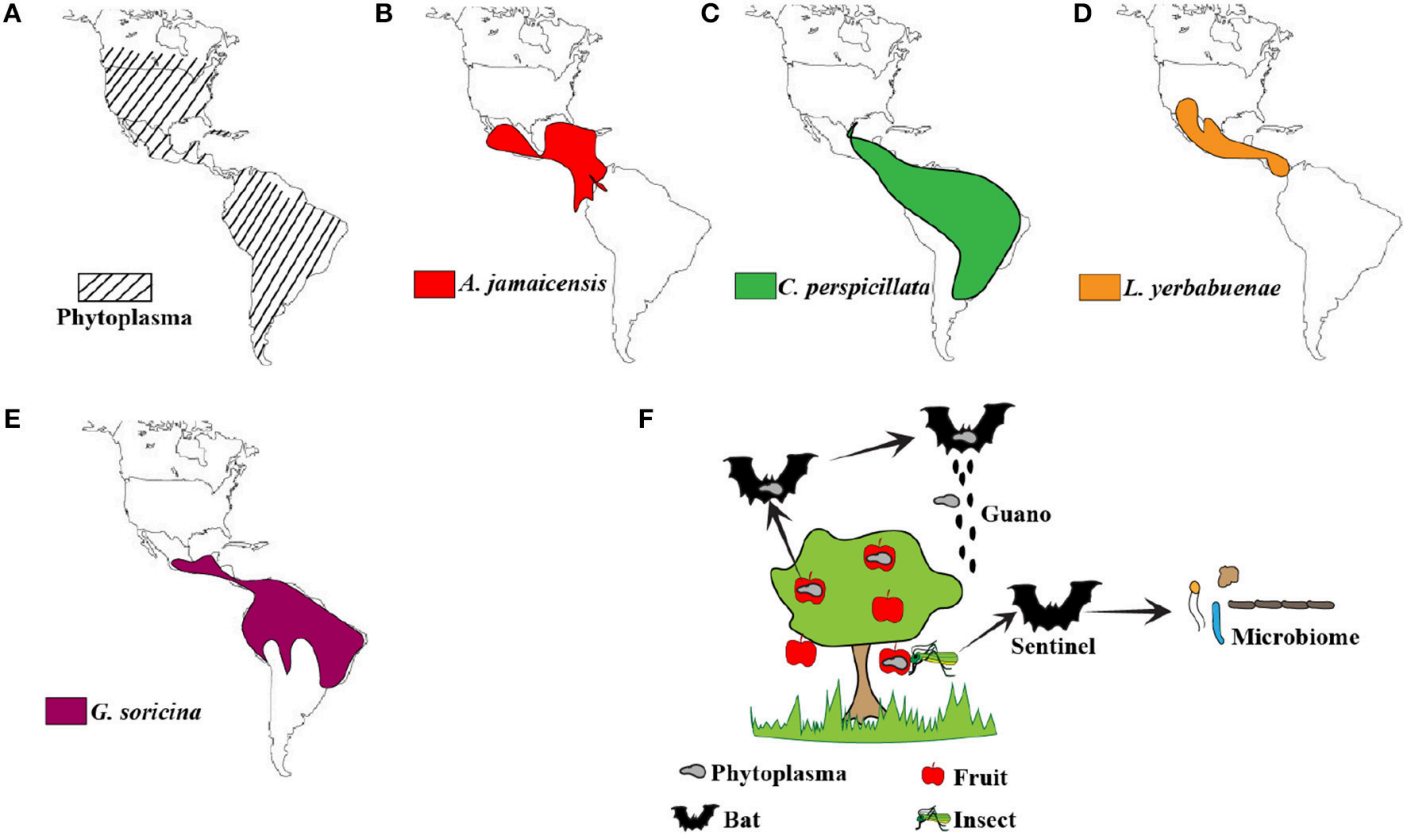
Distribution of phytoplasmas and bats. **(A)** Phytoplasma distribution in the Americas (Lee et al., 2000; Pérez-López et al., 2016). **(B–E)** Geographical distribution of *A. jamaicensis* (Miller et al., 2016)*, C. perspicillata* (Barquez et al., 2015a)*, L. yerbabuenae* (Medellin, 2016), *and G. soricina* (Barquez et al., 2015b; IUCN, 2018) sampled in this study by Carrillo-Araujo et al. **(F)** Schematic representation of the possible role of bats as vectors and sentinels of phytoplasmas. Fruit and nectivorous bats could potentially move phytoplasmas around through guano or seeds that are part of the guano. Alternatively, microbiome analysis of insectivorous bats that feed on fruit eating insects could allow us to monitor phytoplasma prevalence and spread.

Multiple studies have established the role of bats as reservoirs of certain mammalian viruses (Corman et al., 2014; Plowright et al., 2016; Ng and Tan, 2017; Noh et al., 2017; Widagdo et al., 2017). Similar studies are needed for plant pathogens. In this study, the authors analyzed the microbiome of bats using DNA sequencing. The ability to culture *Mollicutes* from bat intestinal samples would identify if these bacteria remain viable while they pass through the harsh environment of the digestive tract. Unfortunately, many of the plant pathogenic *Mollicutes*, including phytoplasmas, are unculturable in axenic media. Although *Mollicutes* are unlikely to replicate in bat gut cells, they could potentially proliferate within the intestinal micro-community. However, this remains to be tested.

Other questions about the possible excretion of viable bacteria through bat guano and the ability to infect plants remain unknown (Figure 1F). Seeds could be part of bat guano, but phytoplasma transmission through seeds has not been confirmed or disproved yet. Alongside birds, bats are capable of true flight. The possibility to deposit phytoplasma-contaminated guano from one area to another and within the same area is high. Aryan et al. showed that phytoplasmas are transmissible through graft (Aryan et al., 2016). Thus, mechanical transmission is another possibility. This form of transmission occurs when feeding animals cause tissue damage in plants, aiding the spread of microorganisms.

While bats are speculated as reservoirs of multiple microbes, this study brings up the possibility of using bat microbiome data as predictors of pathogen spread and prevalence (Figure 1F). Although our knowledge about the bat microbiome is limited, it does provide us with an opportunity to study bats as sentinels of plant pathogens. This opportunity extends to insect-eating bats, since insects such as leafhoppers and planthoppers are known vectors for phytoplasmas (Weintraub and Beanland, 2006; Pérez-López et al., 2018).

Future studies focused on identifying neglected vectors of plant pathogens will elucidate the likely role played by herbivorous wildlife in the dispersal of these microorganisms. Results from such studies will have agricultural policy implications for plant diseases. Phytoplasmas continue to cause losses to local farmers and has an impact on the economy. In our opinion, this study by Carrillo-Araujo et al. that identified *Mollicutes* in the intestinal content of Phyllostomid bats opens up an alternate and intriguing line of investigation in to wildlife vectors and sentinels of plant pathogens.

## Author contributions

AB and EP-L wrote the commentary. KM edited the commentary.

### Conflict of interest statement

The authors declare that the research was conducted in the absence of any commercial or financial relationships that could be construed as a potential conflict of interest.
